# Dorsal bridge plating, volar plating, or both? A retrospective analysis of clinical and radiographic outcomes of intraarticular distal radius fracture fixation techniques

**DOI:** 10.1007/s00590-026-04797-9

**Published:** 2026-06-04

**Authors:** Robin Litten, Anthony Wilson, Matthew Yeager, Robert Rutz, Ryan McIlwain, Katie Frith, Jonathan Quade

**Affiliations:** 1 Department of Orthopaedic Surgery, University of Alabama at Birmingham, Birmingham, USA; 2 Department of Orthopaedic Surgery, University of South Florida, Tampa, USA; 3 Department of Orthopaedic Surgery, Louisiana State University Health Sciences Center Shreveport, Shreveport, USA

**Keywords:** Distal radius fracture, Volar plate, Dorsal bridge plate, Radiographic outcomes

## Abstract

**Purpose:**

Optimal fixation for complex AO/OTA 2R3C intraarticular distal radius fractures remains debated, particularly the role of dorsal bridge plating (DBP) alone or in combination with volar plating (VP). This study compared radiographic outcomes and complications across VP, DBP, and combined VP-DBP constructs.

**Methods:**

A retrospective cohort study was conducted at a single Level I trauma center (2020–2024). Adults with AO/OTA 2R3C distal radius fractures treated with VP alone, DBP alone, or combined VP-DBP and with ≥ 6 months of follow-up were included. Standard radiographic parameters (volar tilt [VT], radial height [RH], radial inclination [RI], and ulnar variance [UV]) were measured immediately postoperatively and at final follow-up. Wrist range of motion (flexion, extension, pronation, supination) at final follow-up was recorded from clinic documentation. Complications included fracture-related infection (FRI), reoperation to promote bone healing, hardware removal (HWR), and unplanned reoperation. Group comparisons used one-way ANOVA and chi-square/Fisher’s exact tests.

**Results:**

A total of 153 patients (mean age: 50 years) were included; 84 (54.9%) underwent VP, 44 (28.8%) DBP, and 25 (16.3%) combined fixation. Immediately postoperatively, DBP achieved greater RI (22.3° versus 19.7° VP versus 18.9° combined) and RH (13.4 mm versus 11.6 mm versus 11.3 mm), while VT was greatest with VP (9.9° versus 8.3° DBP versus 4.5° combined; all *p* < 0.05). At final follow-up, UV, VT, RI, and RH did not differ significantly between constructs, although all groups demonstrated increased UV over time, greatest in the combined cohort. FRI (0-3.6%) and unplanned reoperation (7.8% overall) rates were similar across groups. HWR was more frequent after DBP and combined fixation, reflecting planned implant removal. At final follow-up, VP demonstrated greater wrist flexion and extension compared with DBP and combined fixation (*p* < 0.05), while pronation and supination were similar between groups.

**Conclusion:**

Across fixation strategies for intraarticular distal radius fractures, no single construct demonstrated radiographic superiority at final follow-up, although volar plating was associated with greater flexion and extension at final follow-up. These findings underscore the importance of tailoring fixation strategy to fracture pattern and patient-specific factors when managing complex distal radius fractures.

## Introduction

**Fig. 1:**
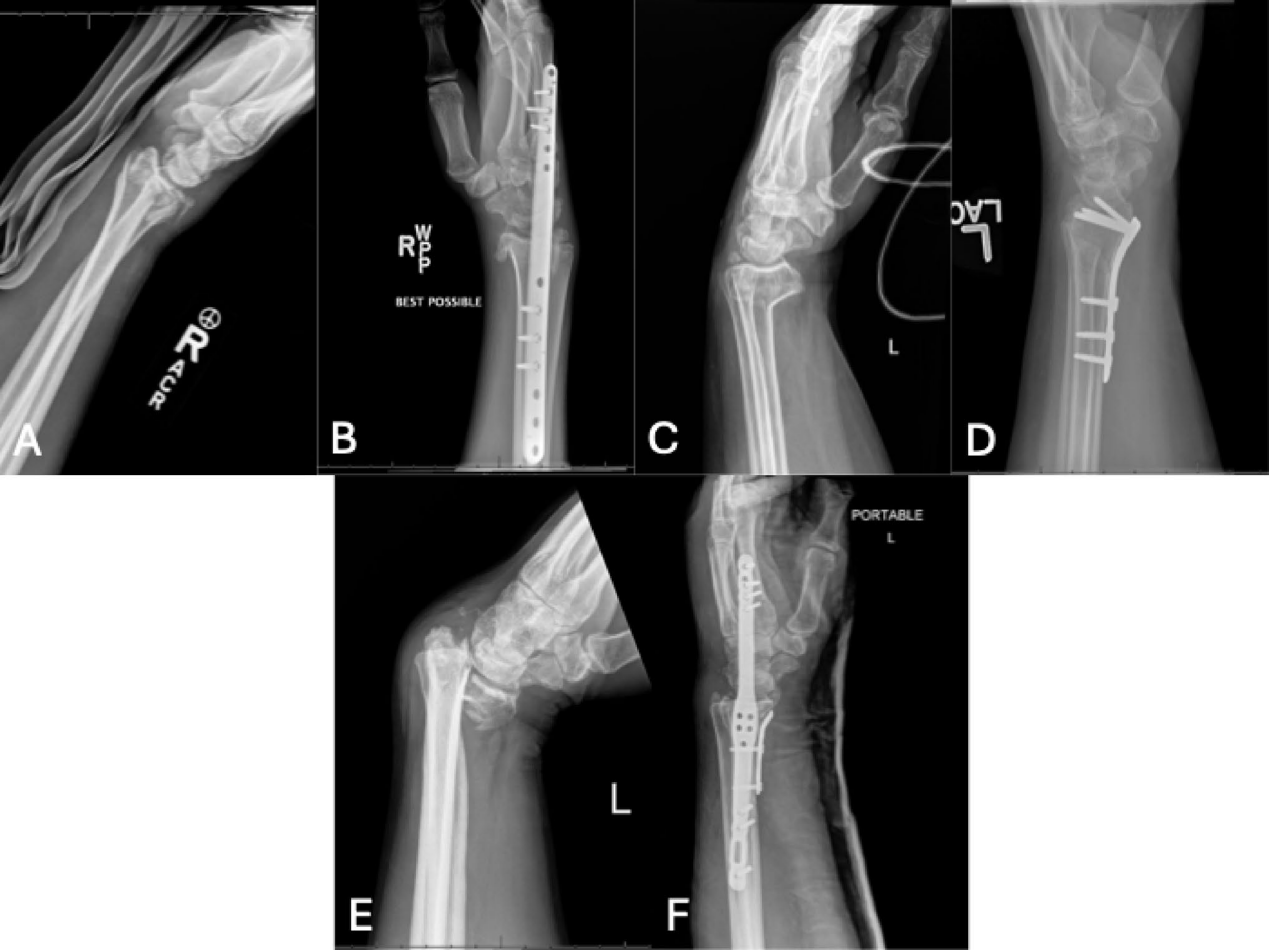
Representative radiographic examples of fixation constructs used for AO/OTA 2R3C distal radius fractures. **A** Injury radiograph of a right distal radius fracture. **B** Postoperative radiograph demonstrating dorsal bridge plate fixation. **C** Injury radiograph of a left distal radius fracture. **D** Postoperative radiograph demonstrating volar plate fixation. **E** Injury radiograph of a left distal radius fracture. **F** Postoperative radiograph demonstrating combined dorsal bridge plate and volar plate fixation

Distal radius fractures account for approximately 17% of fractures, making it the most common fracture of the upper limb with an increasing incidence worldwide [1–6]. Their bimodal distribution reflects high-energy trauma in younger males and low-energy osteoporotic injuries in older females [7–13]. Treatment options include closed reduction and casting, closed reduction and percutaneous fixation, and open reduction internal fixation (ORIF) [14]. Volar plating (VP) is widely used and has become the predominant surgical technique for unstable fractures [15, 16], while dorsal bridge plating (DBP) and combined volar-dorsal constructs are additional options for complex, comminuted patterns (Fig. 1).

Standard radiographic parameters (volar tilt [VT], radial height [RH], radial inclination [RI], and ulnar variance [UV]) are routinely assessed after fixation and have been shown to correlate with wrist function and long-term outcomes [17–20]. However, most data describing these metrics derive from simpler or extra-articular fractures [18–20]. Evidence remains limited regarding how different fixation constructs influence radiographic restoration and maintenance in complex intraarticular fractures such as AO/OTA type 2R3C [21–23].

The purpose of this study was to compare postoperative VT, RH, RI, and UV, as well as complication rates, among patients with AO/OTA type 2R3C intraarticular distal radius fractures treated with VP alone, DBP alone, or combined VP-DBP fixation (Fig. 1). The authors hypothesized that DBP would provide superior maintenance of RH and RI over time compared with VP or combined fixation.

## Methods

### Study design, setting, and participants

Following institutional review board approval, a retrospective review was conducted to identify patients aged 18 years or older who sustained AO/OTA type 2R3C distal radius fractures from a single Level I trauma center from 2020 to 2024. Patients were identified using Current Procedural Terminology (CPT) codes 25,608 and 25,609.

Patients were included if they sustained AO/OTA type 2R3C distal radius fractures managed operatively with a VP alone, DBP alone, or combined VP-DBP fixation (Fig. 1). Patients were excluded if they were under 18 years of age, had less than six months of clinical follow-up, or if their most recent radiographs were obtained fewer than six months postoperatively. Additional exclusions were nonoperative treatment, surgical procedures other than DBP or VP, and fractures classified as AO/OTA type 2R3A or 2R3B. Patients with inadequate anteroposterior (AP) and lateral radiographs that could compromise the accuracy of anatomic measurements were also excluded (*n* = 10). Construct selection (VP, DBP, or combined fixation) was determined at the discretion of the treating surgeon based on fracture pattern, soft-tissue considerations, and surgeon preference.

Patient demographics, including age, sex, race, body mass index (BMI), tobacco use, intravenous drug use (IVDU), and alcohol use were recorded. Comorbidities such as diabetes mellitus (DM), hypertension (HTN), and chronic obstructive pulmonary disease (COPD), as well as American Society of Anesthesiologists (ASA) scores, were collected. Injury characteristics included laterality, mechanism of injury, open versus closed injury, Gustilo-Anderson classification for open fractures, AO/OTA subclassification (2R3C1, 2R3C2, or 2R3C3), polytrauma status, and presence of an ipsilateral upper extremity injury. Surgical information was also recorded, including fixation construct used (DP, VP, or combined VP-DBP fixation), operative time, and estimated blood loss (EBL).

Postoperative complications were noted, including fracture-related infection (FRI), reoperation to promote bone healing (RPBH), hardware removal (HWR), and unplanned reoperation. FRI was defined using the criteria established by Metsemakers et al. in 2018 [24]. RPBH was defined as any secondary procedure performed to facilitate fracture healing, including bone grafting, revision fixation, or biologic or soft-tissue augmentation, in the absence of infection or hardware failure as the primary indication [25, 26]. Functional outcomes were assessed using wrist range of motion (ROM) documented at the latest clinical follow-up (≥ 6 months postoperatively), including flexion, extension, pronation, and supination.

HWR was defined as any documented surgical removal of fixation hardware, whether planned or in response to symptoms such as pain, tendon irritation, or soft tissue impingement. Unplanned reoperation was defined as any secondary surgical procedure not part of the initial operative plan, undertaken to address a postoperative complication, excluding planned hardware removal when documented. All variables were obtained through a retrospective review of the electronic medical record.

### Radiographic measurements

Radiographs at immediate postoperative and final clinical follow-up were reviewed. The primary outcomes were differences in VT, RH, RI, and UV between patients who were treated with VP, DBP, or combined VP-DBP fixation. All measurements were recorded using AP and lateral radiographs. Normal anatomic values for VT, RH, RI, and UV were defined based on the parameters established by Perugia et al. [20].

**Fig. 2:**
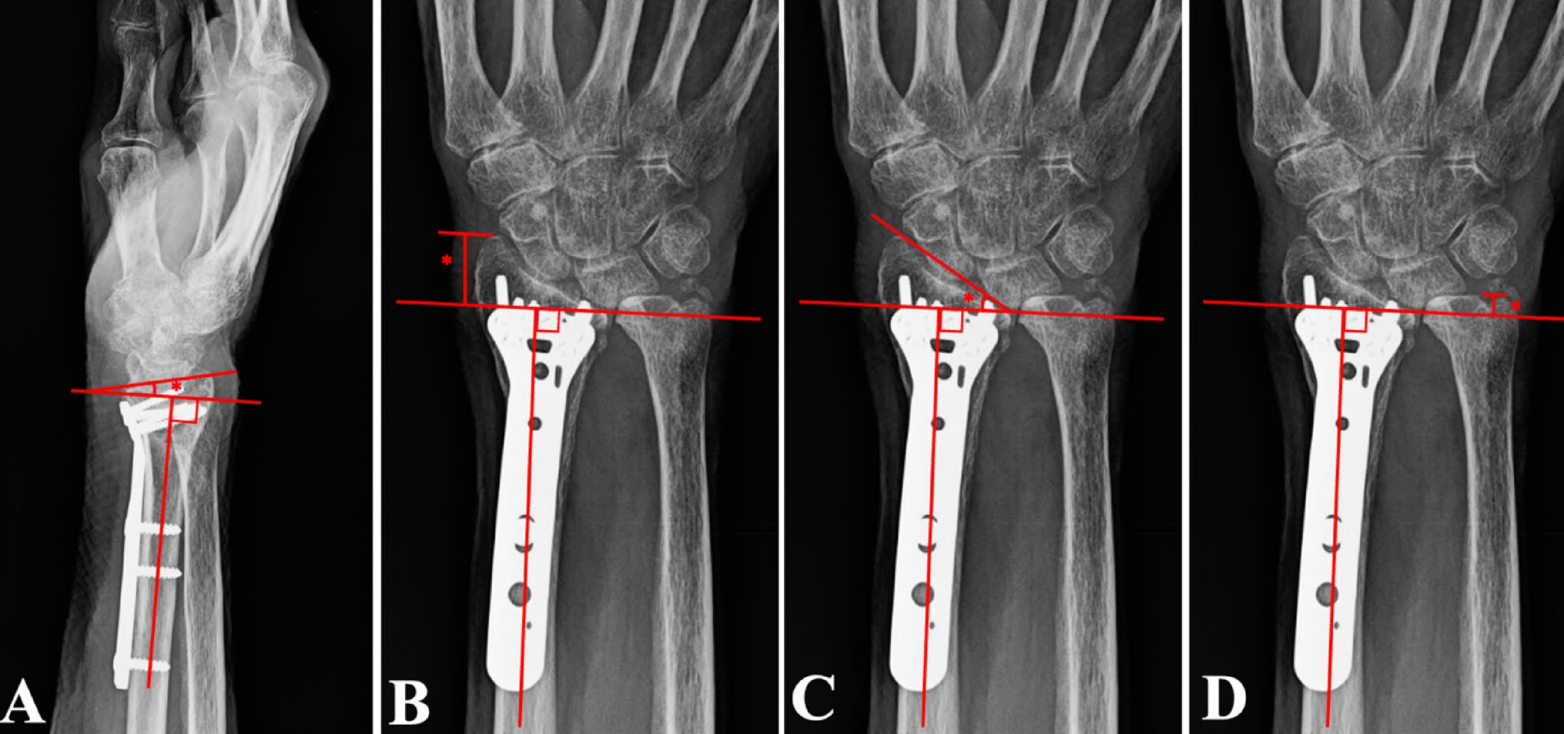
Standard radiographic measurements used to evaluate distal radius alignment: **A** volar tilt, **B** radial height, **C** radial inclination, and **D** ulnar variance

VT was measured on a lateral wrist radiograph by first drawing a line parallel to the longitudinal axis of the radial shaft (Fig. 2A). A second line was drawn connecting the most distal points of the dorsal and volar rims of the distal radial articular surface. The angle between these two lines represented the VT (Fig. 2A).

RH, RI, and UV were measured on an AP radiograph of the wrist. To measure RH, a line was drawn parallel to the longitudinal axis of the radial shaft (Fig. 2B). A second line, perpendicular to the first, was drawn across the distal articular surface at the level of the radioulnar joint (Fig. 2B). RH was defined as the vertical distance from this perpendicular line to the apex of the radial styloid (Fig. 2B).

RI was measured as the angle between a line connecting the radial styloid and the ulnar corner of the distal radius and a line perpendicular to the radial shaft (Fig. 2C). UV was measured as the vertical distance between the perpendicular reference line at the radioulnar joint and the most distal point of the ulnar articular surface (Fig. 2D). Neutral describes the radius and ulna at the same level, positive describes the ulna more distally, and negative describes the ulna more proximally. All radiographic measurements are illustrated and summarized in Fig. 2. These measurements were recorded by a single reviewer and verified by a fellowship-trained orthopaedic trauma surgeon.

### Statistical analyses

Descriptive statistics were generated to characterize the study population. Categorical variables are presented as counts and percentages and were compared using chi-square or Fisher’s exact test, as appropriate. Continuous variables were assessed for approximate normality prior to analysis. Continuous variables are reported as means with standard deviations and were compared either using independent t-tests or one-way analysis of variance (ANOVA), as appropriate. Comparison of continuous variables across the three fixation groups (DBP, VP and combined VP-DBP) were performed using one-way ANOVA. When overall ANOVA results were significant, pairwise post-hoc comparisons were conducted to calculate mean differences with corresponding 95% confidence intervals.

Immediate postoperative and latest follow-up radiographic parameters were compared across fixation constructs using one-way ANOVA with post-hoc pairwise testing. To evaluate longitudinal change in radiographic alignment within each fixation group, postoperative and latest follow-up measurements were compared using paired t-tests, and results are reported as mean differences with 95% confidence intervals. Continuous variables were assessed for approximate normality prior to analysis to confirm the appropriateness of parametric testing. The threshold for statistical significance was defined as a two-sided *p*-value of ≤ 0.05. All statistical analyses were conducted using IBM SPSS Statistics version 29.0.2.0.

## Results

A total of 153 patients with AO/OTA type 2R3C distal radius fractures were included in the study, including 44 treated with DBP, 84 with VP, and 25 with combined fixation. Baseline demographic and clinical characteristics stratified by fixation construct are presented in Table 1. The mean age of this cohort was 50.0 years (range: 18–86 years), and the average follow-up duration was 310.3 days (range: 180–957 days). The cohort was 51.0% male, and most patients identified as white (76.5%). The mean BMI of this cohort was 30.4 kg/m^2^ (range: 16.7–62.2 kg/m^2^). Hypertension (33.3%), alcohol use (35.3%), and tobacco use (24.8%) were the most common comorbidities, while diabetes (9.8%), COPD (6.5%), and intravenous drug use (2.6%) were less frequently reported. ASA classification scores were primarily ASA II (28.8%) and ASA III (60.8%) (Table 1).

**Table 1:** Demographic and clinical characteristics of patients with intraarticular distal radius fractures, stratified by fixation construct

Variable	Total (*n* = 153)	DBP (*n* = 44)	VP (*n* = 84)	DBP and VP (*n* = 25)	*p*-value
Age (years), mean (SD)	50.0 (17.5)	53.2 (18.9)	48.3 (17.6)	52.1 (14.5)	0.280
Follow-up (days), mean (SD)	310.3 (135.2)	293.2 (106.8)	331.2 (158.9)	270.2 (64.7)	**0.023**
*Sex, n (%)*					
Male	78 (51.0)	26 (59.1)	39 (46.4)	13 (52.0)	0.394
Female	75 (49.0)	18 (40.9)	45 (53.6)	12 (48.0)	
*Race, n (%)*					
White	117 (76.5)	34 (79.1)	61 (72.6)	22 (88.0)	0.096
Black	26 (17.0)	5 (11.6)	20 (23.8)	1 (4.0)	
Other	9 (5.9)	4 (9.3)	3 (3.6)	2 (8.0)	
BMI (kg/m^2^), mean (SD)	30.4 (7.7)	29.6 (8.4)	30.8 (7.4)	30.4 (7.3)	0.712
Diabetes, n (%)	15 (9.8)	4 (9.1)	7 (8.3)	4 (16.0)	0.518
Hypertension, n (%)	51 (33.3)	16 (36.4)	23 (27.7)	12 (48.0)	0.152
Tobacco use, n (%)	38 (24.8)	12 (27.3)	18 (21.4)	8 (32.0)	0.509
Alcohol use, n (%)	54 (35.3)	14 (31.8)	26 (31.0)	14 (56.0)	0.060
IVDU, n (%)	4 (2.6)	1 (2.3)	3 (3.6)	0	0.609
COPD, n (%)	10 (6.5)	6 (13.6)	2 (2.4)	2 (8.0)	**0.048**
*ASA score, n (%)*					
I	9 (5.9)	2 (4.5)	7 (8.3)	0	0.147
II	44 (28.8)	11 (25.0)	25 (29.8)	8 (32.0)	
III	93 (60.8)	26 (59.1)	50 (59.5)	17 (68.0)	
IV	7 (4.6)	5 (11.4)	2 (2.4)	0	

Bolded p-values indicate statistical signifi cance at p ≤ 0.05.

DBP = dorsal bridge plate, VP = volar plate, DBP and VP = combined dorsal bridge plate and volar plate fixation, SD = standard deviation, kg/m^2^ = kilograms per meter squared, BMI = body mass index, IVDU = intravenous drug use, COPD = chronic obstructive pulmonary disease, ASA = American Society of Anesthesiologists

Injury characteristics and operative variables stratified by fixation construct are presented in Table 2. Overall, VP was the most common fixation technique (54.9%), followed by dorsal bridge plating (28.8%) and combined dorsal and volar plating (16.3%) (Table 2). Polytrauma was identified among 62.1% of cases, and 20.3% of patients in this cohort sustained an ipsilateral upper extremity injury (Table 2). Open injuries occurred in 15.0% of patients, with the majority classified as Gustilo-Anderson type I (8.5%) or II (5.9%) (Table 2). The most common mechanisms of injury were motor vehicle collisions (37.3%), falls from height greater than 10 feet (20.3%), and ground-level falls (20.3%) (Table 2). Mechanism of injury differed significantly between constructs (*p* = 0.009), with falls from height over 10 feet more frequently observed in the combined fixation cohort (Table 2). AO/OTA subclassification included 37 (24.2%) 2R3C1 fractures, 86 (56.2%) 2R3C2 fractures, and 30 (19.6%) 2R3C3 fractures, with no significant difference in subclassification distribution across fixation constructs (*p* = 0.505) (Table 2).

**Table 2:** Injury profile and surgical characteristics among patients with intraarticular distal radius fractures, stratified by fixation construct

Variable	Total (*n* = 153)	DBP (*n* = 44)	VP (*n* = 84)	DBP and VP (*n* = 25)	*p*-value
*Laterality, n (%)*					
Left	79 (51.6)	27 (61.4)	38 (45.2)	14 (56.0)	0.198
Right	74 (48.4)	17 (38.6)	46 (54.8)	11 (44.0)	
Ipsilateral UE injury, n (%)	31 (20.3)	9 (20.5)	18 (21.4)	4 (16.0)	0.838
Polytrauma, n (%)	95 (62.1)	30 (68.2)	49 (58.3)	16 (64.0)	0.539
OR time (min), mean (SD)	141.3 (71.3)	147.7 (87.3)	133.6 (68.8)	156.3 (42.3)	0.309
EBL (mL), mean (SD)	94.8 (171.6)	154.9 (258.7)	68.1 (102.9)	77.1 (140.0)	0.121
Open injury, n (%)	23 (15.0)	7 (15.9)	13 (15.5)	3 (12.0)	0.896
*Gustilo-Anderson type, n (%)*					
I	13 (8.5)	3 (42.9)	8 (61.5)	2 (66.7)	0.745
II	9 (5.9)	4 (57.1)	4 (30.8)	1 (33.3)	
III	1 (0.7)	0	1 (7.7)	0	
*Mechanism of injury, n (%)*					
MVC	57 (37.3)	18 (40.9)	35 (41.7)	4 (16.0)	**0.009**
MCC	16 (10.5)	6 (13.6)	8 (9.5)	2 (8.0)	
FFH > 10ft	31 (20.3)	8 (18.2)	10 (11.9)	13 (52.0)	
FFH < 10ft	6 (3.9)	0	6 (7.1)	0	
GLF	31 (20.3)	10 (22.7)	17 (20.2)	4 (16.0)	
ATV	4 (2.6)	1 (2.3)	2 (2.4)	1 (4.0)	
Other	8 (5.2)	1 (2.3)	6 (7.1)	1 (4.0)	
*AO/OTA class*					
2R3C1	37 (24.2)	11 (25.0)	20 (23.8)	6 (24.0)	0.505
2R3C2	86 (56.2)	22 (50.0)	47 (56.0)	17 (68.0)	
2R3C3	30 (19.6)	11 (25.0)	17 (20.2)	2 (8.0)	

Bolded p-values indicate statistical signifi cance at p ≤ 0.05.

DBP = dorsal bridge plate, VP = volar plate, DBP and VP = combined dorsal bridge plate and volar plate fixation, SD = standard deviation, UE = upper extremity, OR = operating room, min = minutes, EBL = estimated blood loss, ft = feet, mL = milliliters, MVC = motor vehicle collision, MCC = motorcycle collision, FFH = fall from height, GLF = ground level fall, ATV = all-terrain vehicle accident

Postoperative complications and final ROM are presented in Table 3. The rate of FRI was 2.3% in the DBP group, 3.6% in the VP group, and 0% in the combined fixation group (*p* = 0.609) (Table 3). Unplanned reoperation was documented in 6.8% of DBP, 10.7% of VP, and 0% of combined fixation patients (*p* = 0.207) (Table 3). HWR was significantly more common in the DBP (45.5%) and combined (40.0%) groups compared to the VP group (8.3%) (*p* < 0.001). Of note, however, HWR is typically planned as part of the treatment pathway following DBP fixation (Table 3). Furthermore, these values represent HWR documented during the available follow-up period and may not reflect the final proportion of patients who ultimately underwent planned bridge plate removal.

**Table 3:** Postoperative outcomes by fixation construct among patients with intraarticular distal radius fractures

Variable	DBP (*n* = 44)	VP (*n* = 84)	DBP and VP (*n* = 25)	*p*-value
FRI, n (%)	1 (2.3)	3 (3.6)	0 (0)	0.609
Unplanned reoperation, n (%)	3 (6.8)	9 (10.7)	0 (0)	0.207
RPBH, n (%)	5 (11.4)	4 (4.8)	1 (4.0)	0.305
HWR, n (%)	20 (45.5)	7 (8.3)	10 (40.0)	**< 0.001**
Flexion, (degrees), mean (SD)	41.7 (19.2)	54.7 (17.2)	44.8 (11.3)	**0.012**
Extension, (degrees), mean (SD)	41.5 (23.6)	54.7 (20.6)	39.0 (16.8)	**0.018**
Pronation, (degrees), mean (SD)	76.9 (13.2)	71.7 (13.1)	74.6 (12.7)	0.472
Supination, (degrees), mean (SD)	66.2 (21.1)	70.8 (15.4)	73.1 (7.7)	0.514

Bolded p-values indicate statistical signifi cance at p ≤ 0.05.

DBP = dorsal bridge plate, VP = volar plate, DBP and VP = combined dorsal bridge plate and volar plate fixation, RPBH = reoperation to promote bone healing, HWR = hardware removal, FRI = fracture-related infection

Flexion and extension differed significantly by fixation construct: patients treated with VP demonstrated greater flexion (54.7°) compared with DBP (41.7°) and combined fixation (44.8°) (*p* = 0.012) (Table 3). Similarly, patients treated with VP demonstrated greater extension (54.7°) compared with DBP (41.5°) and combined fixation (39.0°) (*p* = 0.018) (Table 3). Pronation and supination were similar across constructs (*p* = 0.472 and *p* = 0.514, respectively) (Table 3).

Immediate postoperative radiographic outcomes by fixation construct are summarized in Table 4. UV did not differ significantly among the three fixation groups (*p* = 0.576) (Table 4). However, patients treated with DBP exhibited lower VT (8.3°) compared with those treated with VP (9.9°), while the combined DBP- VP group demonstrated the lowest VT overall (4.5°; *p* = 0.022) (Table 4). RI and RH also varied by construct, with the DBP group demonstrating greater values (22.3° and 13.4 mm, respectively) than the VP group (19.7° and 11.6 mm) and the combined fixation group (18.9° and 11.3 mm) (*p* = 0.013 and *p* = 0.036, respectively) (Table 4).

**Table 4:** Immediate postoperative radiographic outcomes by fixation construct among patients with intraarticular distal radius fractures

Variable	DBP, mean (SD)	VP, mean (SD)	DBP + VP, mean (SD)	*p*-value
Ulnar variance (mm)	1.1 (3.0)	0.7 (2.4)	0.2 (3.4)	0.576
Volar tilt (°)	8.3 (9.1)	9.9 (7.2)	4.5 (9.3)	**0.022**
Radial inclination (°)	22.3 (5.8)	19.7 (4.7)	18.9 (5.0)	**0.013**
Radial height (mm)	13.4 (4.4)	11.6 (2.6)	11.3 (2.5)	**0.036**

Bolded p-values indicate statistical signifi cance at p ≤ 0.05.

SD = standard deviation, DBP = dorsal bridge plate, VP = volar plate, DBP + VP = combined dorsal bridge plate and volar plate fixation, ° = degrees, mm = millimeters

Pairwise comparisons of immediate postoperative radiographic parameters are presented in Table 5. VP fixation achieved significantly greater VT compared with combined DBP-VP fixation (mean difference 5.36°, 95% CI 0.82 to 9.90; *p* = 0.016) (Table 5). RI differed significantly between DBP and VP (mean difference 2.51°, 95% CI 0.24 to 4.79; *p* = 0.027) and between DBP and combined fixation (mean difference 3.34°, 95% CI 0.25 to 6.43; *p* = 0.031) (Table 5). RH was found to be higher in the DBP group compared with VP (mean difference 1.81 mm, 95% CI 0.05 to 3.57; *p* = 0.043) and combined fixation (mean difference 2.11 mm, 95% CI 0.09 to 4.15; *p* = 0.039) (Table 5).

**Table 5:** Immediate postoperative radiographic comparisons across fixation constructs among patients with intraarticular distal radius fractures

Variable	Mean difference (95% CI)	*p*-value
*Ulnar variance (mm)*		
DBP versus VP	0.40 (− 0.86 to 1.65)	0.734
DBP versus both	0.83 (− 1.14 to 2.79)	0.568
VP versus both	0.43 (− 1.34 to 2.21)	0.823
*Volar tilt (°)*		
DBP versus VP	1.55 (− 5.27 to 2.17)	0.587
DBP versus both	3.81 (− 1.21 to 8.84)	0.174
VP versus both	5.36 (0.82 to 9.90)	**0.016**
*Radial inclination (°)*		
DBP versus VP	2.51 (0.24 to 4.79)	**0.027**
DBP versus both	3.34 (0.25 to 6.43)	**0.031**
VP versus both	0.82 (− 1.97 to 3.62)	0.764
*Radial height (mm)*		
DBP versus VP	1.81 (0.05 to 3.57)	**0.043**
DBP versus both	2.11 (0.09 to 4.15)	**0.039**
VP versus both	0.31 (− 1.12 to 1.75)	0.856

Bolded p-values indicate statistical signifi cance at p ≤ 0.05.

CI = confidence interval, DBP = dorsal bridge plate, VP = volar plate, both = combined dorsal bridge plate and volar plate fixation, ° = degrees, mm = millimeters

Radiographic parameters at latest follow-up are presented in Table 6. At final follow-up, there were no statistically significant differences in UV, VT, RI, or RH between the three fixation groups (Table 6). However, DBP continued to demonstrate the highest RI and RH (22.4° and 13.0 mm). Furthermore, while not statistically significant, the VP group also demonstrated the highest VT (9.7°; *p* = 0.069). Pairwise comparisons (Table 7) revealed no statistically significant differences between groups at latest follow-up.

**Table 6:** Radiographic outcomes at latest follow-up by fixation construct among patients with intraarticular distal radius fractures

Variable	DBP, mean (SD)	VP, mean (SD)	DBP + VP, mean (SD)	*p*-value
Ulnar variance (mm)	2.4 (3.9)	1.4 (2.3)	2.0 (3.3)	0.235
Volar tilt (°)	9.6 (9.6)	9.7 (7.6)	5.2 (10.4)	0.069
Radial inclination (°)	22.4 (6.9)	21.5 (4.5)	21.5 (6.6)	0.749
Radial height (mm)	13.0 (3.7)	11.7 (2.7)	12.4 (3.6)	0.114

SD = standard deviation, DBP = dorsal bridge plate, VP = volar plate, DBP + VP = combined dorsal bridge plate and volar plate fixation, ° = degrees, mm = millimeters

**Table 7:** Latest follow-up radiographic comparisons across fixation constructs among patients with intraarticular distal radius fractures

Variable	Mean difference (95% CI)	*p*-value
*Ulnar variance (mm)*		
DBP versus VP	1.05 (− 0.49 to 2.58)	0.239
DBP versus both	0.48 (− 1.66 to 2.62)	0.852
VP versus both	− 0.57 (− 2.31 to 1.18)	0.706
*Volar tilt (°)*		
DBP versus VP	− 0.16 (− 4.00 to 3.67)	0.994
DBP versus both	4.33 (− 0.83 to 9.49)	0.119
VP versus both	4.49 (− 0.20 to 9.18)	0.064
*Radial inclination (°)*		
DBP versus VP	0.86 (− 1.90 to 3.62)	0.736
DBP versus both	0.94 (− 3.10 to 4.99)	0.841
VP versus both	0.08 (− 3.37 to 3.54)	0.998
*Radial height (mm)*		
DBP versus VP	1.32 (− 0.20 to 2.83)	0.102
DBP versus both	0.64 (− 1.58 to 2.87)	0.764
VP versus both	− 0.67 (− 2.60 to 1.26)	0.673

CI = confidence interval, DBP = dorsal bridge plate, VP = volar plate, both = combined dorsal bridge plate and volar plate fixation, ° = degrees, mm = millimeters

Changes in radiographic alignment from the immediate postoperative period to latest follow-up are summarized in Table 8. UV increased significantly across all fixation constructs, with mean changes of 1.3 mm (95% CI 0.22–2.54) for DBP, 0.7 mm (95% CI 0.25 to 1.20) for VP, and 1.72 mm (95% CI 0.86 to 2.59) for combined fixation (*p* = 0.021, 0.003, and < 0.001, respectively) (Table 8). No significant changes in VT were observed for any construct (Table 8). RI decreased significantly in the VP group (-1.79°, 95% CI − 2.82 to − 0.75; *p* < 0.001) and in the combined fixation group (-3.10°, 95% CI − 5.19 to − 1.00; *p* = 0.006), while remaining stable in the DBP group (-0.18°, 95% CI − 1.73 to 1.37; *p* = 0.820) (Table 8). RH decreased significantly in the combined DBP-VP group (-1.43 mm, 95% CI − 2.42 to − 0.45; *p* = 0.006), with no significant changes identified for DBP or VP fixation (Table 8).

**Table 8:** Differences in radiographic parameters from postoperative to latest follow-up by fixation construct

Measure	Mean difference (95% CI)	*p*-value
*Ulnar variance (mm)*		
DBP	1.3 (0.22 to 2.54)	**0.021**
VP	0.7 (0.25 to 1.20)	**0.003**
Both	1.72 (0.86 to 2.59)	**< 0.001**
*Volar tilt (°)*		
DBP	− 1.17 (− 3.65 to 1.30)	0.343
VP	0.25 (− 1.37 to 1.42)	0.972
Both	− 1.03 (− 4.13 to 2.07)	0.498
*Radial inclination (°)*		
DBP	− 0.18 (− 1.73 to 1.37)	0.820
VP	− 1.79 (− 2.82 to − 0.75)	**< 0.001**
Both	− 3.10 (− 5.19 to − 1.00)	**0.006**
*Radial height (mm)*		
DBP	0.23 (− 0.88 to 1.33)	0.681
VP	− 0.12 (− 0.57 to 0.34)	0.611
Both	− 1.43 (− 2.42 to − 0.45)	**0.006**

Bolded p-values indicate statistical signifi cance at p ≤ 0.05.

CI = confidence interval, DBP = dorsal bridge plate, VP = volar plate, both = combined dorsal bridge plate and volar plate fixation, ° = degrees, mm = millimeters

## Discussion

In this retrospective cohort of patients with AO/OTA type 2R3C intraarticular distal radius fractures, fixation construct influenced early but not long-term radiographic alignment. DBP achieved greater initial restoration of RH and RI, whereas VP demonstrated greater VT. By latest follow-up, however, radiographic differences were no longer statistically significant, and all groups demonstrated progressive loss of alignment, including increased UV, which was most pronounced in the combined fixation cohort. Postoperative complications were comparable across constructs, with expected higher HWR rates in the DBP and combined groups reflecting planned implant removal. At latest follow-up, VP was associated with greater wrist flexion and extension compared with DBP and combined fixation, while pronation and supination were similar between groups. Taken together, these findings suggest that no single construct demonstrated clear radiographic superiority at final follow-up, highlighting that fixation strategy should be tailored to fracture characteristics and patient-specific factors.

The rates of FRI observed in this study (0% to 3.6%) are comparable to what is currently reported in the literature on distal radius fractures (1–5%) [27–29]. Additionally, the overall unplanned reoperation rate in this cohort (7.8%; 12/153) was lower than the 24% reported by Stam et al. in a series of 33 patients, but higher than the rates reported by McEntee et al. (1.5%) and Schindelar et al. (0.6%) [30–32]. Although HWR was significantly more common in the DBP (45.5%) and combined fixation (40.0%) groups, this largely reflects planned implant removal inherent to DBP treatment rather than postoperative complication and should therefore not be interpreted as equivalent to unplanned reoperation. Collectively, these findings suggest that each construct can be used safely with comparable postoperative risk.

On immediate postoperative radiographs, DBP fixation achieved significantly greater RI and RH compared with VP and combined constructs, whereas VP fixation demonstrated the greatest VT. These findings differ from Carroll et al. [33], who reported higher RI and RH with VP fixation and no differences in VT, and from Said et al. [34], who found no differences in RI or RH at final follow-up but observed greater VT with VP. In contrast, Calem et al. [22] reported comparable radiographic outcomes between DBP fixation alone and DBP with concomitant VP for comminuted intraarticular distal radius fractures, suggesting limited incremental radiographic benefit of combined constructs. Similarly, Gruenberger et al. demonstrated that supplemental fixation used with a concomitant DBP may be employed in complex intraarticular fracture patterns requiring additional stability, although the overall clinical advantage of these more complex constructs remains uncertain [35]. In the present cohort, the early differences in RH, RI, and VT were no longer statistically significant by latest follow-up. Collectively, the variation across studies and the diminishing radiographic differences over time suggest that multiple fixation constructs can achieve acceptable alignment when appropriately selected.

In addition to radiographic outcomes, differences in wrist ROM were observed across fixation constructs. Patients treated with VP demonstrated significantly greater flexion and extension at final follow-up compared with DBP and combined fixation, while forearm rotation was similar across groups. Prior comparative studies have reported similar findings. For example, Carroll et al. [33] demonstrated greater wrist and forearm ROM following volar locking plate fixation compared with DBP among patients with complete articular distal radius fractures. More recently, Carroll et al. also reported significantly greater flexion, extension, pronation, supination, radial deviation, ulnar deviation, and grip strength following volar locked plating compared with DBP at 6-month follow-up in AO/OTA 2R3C fractures, with corresponding improvements in PROMIS upper extremity and physical function scores [36]. These findings are consistent with the present study, in which VP was associated with greater flexion and extension at final follow-up. Additionally, a systematic review of DBP has noted mild reductions in wrist motion compared with VP constructs [21]. However, the clinical significance of these differences remains uncertain, particularly given the absence of validated patient-reported outcome measures or strength testing in this cohort.

All fixation groups demonstrated a significant increase in UV over time, with the largest change occurring in the combined fixation cohort. As Johnson et al. note, apparent increases in UV may reflect alterations in articular tilt rather than true radial shortening, underscoring the need for careful interpretation of postoperative radiographs [37]. The VP and combined groups also demonstrated decreases in RI, and the combined group exhibited a significant loss of RH, patterns consistent with prior reports of gradual RH decline after VP fixation, particularly in patients with reduced bone quality [38]. Collectively, these longitudinal changes highlight that some degree of alignment change is expected regardless of construct and reinforce the importance of matching fixation strategy to fracture severity and patient-specific factors such as bone quality.

### Limitations

This study has limitations that should be considered when interpreting these findings, including those inherent to its retrospective design. This study was conducted at a single institution, which may limit generalizability to other settings with different patient populations, clinical protocols, or distal radius fracture management practices. Furthermore, all radiographic measurements were performed by a single reviewer (an orthopaedic trauma research fellow) and were subsequently verified by a fellowship-trained orthopaedic trauma surgeon to ensure consistency and minimize variability; however, the accuracy of these measurements may still have been influenced by the investigator’s skill and experience. Additionally, formal inter- or intra-observer reliability testing for radiographic measurements was not performed. Although measurements were standardized and verified by a fellowship-trained orthopaedic trauma surgeon, variability inherent to radiographic assessment may still influence measurement precision.

Construct selection was influenced by surgeon preference and fracture severity; therefore, the fixation groups may not represent directly comparable injury patterns. Consequently, observed differences between constructs could reflect underlying variation in fracture complexity rather than true differences in implant performance. Additionally, although all fractures in this cohort were classified as AO/OTA 2R3C and were further subclassified as 2R3C1, 2R3C2, or 2R3C3, this designation still encompasses a broad spectrum of fracture severity and articular comminution. Because detailed measures of severity (e.g., degree of comminution, metaphyseal defect size, or articular impaction) were not systematically captured, residual differences in injury complexity between groups may persist despite using the same OTA classification.

Given the relatively small size of the DBP and combined fixation cohorts, multivariable adjustment or propensity score matching was not performed due to concerns regarding model instability and loss of statistical power. The limited number of patients in the combined fixation cohort may also reduce statistical power to detect differences in less frequent outcomes such as postoperative complications. Although wrist ROM was captured at latest follow-up, patient-reported outcome measures and grip strength were not consistently available and therefore could not be analyzed. Additionally, although a minimum follow-up of six months was required to ensure adequate time for fracture healing, follow-up duration varied widely and differed significantly between groups, which may influence the assessment of longer-term outcomes such as range of motion recovery or delayed hardware removal. As such, these findings should be interpreted with caution. Future studies with larger cohorts and standardized follow-up intervals are warranted to better assess the long-term clinical implications of these radiographic differences and determine whether certain fixation strategies confer superior functional outcomes.

## Conclusion

Across fixation strategies for AO/OTA 2R3C distal radius fractures, no single construct demonstrated radiographic superiority at final follow-up. Although early differences were observed, these diminished over time, and complication rates remained comparable across groups. Volar plating was associated with greater wrist flexion and extension at final follow-up, while forearm rotation was similar across constructs. These findings underscore the importance of tailoring fixation strategy to fracture pattern and patient-specific factors when managing complex distal radius fractures.

## Data Availability

No datasets were generated or analysed during the current study.
